# Protective Effect of *Momordica charantia* Fruit Extract on Hyperglycaemia-Induced Cardiac Fibrosis

**DOI:** 10.1155/2014/429060

**Published:** 2014-10-13

**Authors:** Razif Abas, Faizah Othman, Zar Chi Thent

**Affiliations:** ^1^Department of Human Anatomy, Faculty of Medicine and Health Science, Universiti Putra Malaysia, 43400 Serdang, Malaysia; ^2^Department of Anatomy, Faculty of Medicine, Universiti Kebangsaan Malaysia, Jalan Raja Muda Abd Aziz, 50300 Kuala Lumpur, Malaysia

## Abstract

In diabetes mellitus, cardiac fibrosis is characterized by increase in the deposition of collagen fibers. The present study aimed to observe the effect of *Momordica charantia* (*MC*) fruit extract on hyperglycaemia-induced cardiac fibrosis. Diabetes was induced in the male Sprague-Dawley rats with a single intravenous injection of streptozotocin (STZ). Following 4 weeks of STZ induction, the rats were subdivided (*n* = 6) into control group (Ctrl), control group treated with *MC* (Ctrl-*MC*), diabetic untreated group (DM-Ctrl), diabetic group treated with *MC* (DM-*MC*), and diabetic group treated with 150 mg/kg of metformin (DM-Met). Administration of *MC* fruit extract (1.5 g/kg body weight) in diabetic rats for 28 days showed significant increase in the body weight and decrease in the fasting blood glucose level. Significant increase in cardiac tissues superoxide dismutase (SOD), glutathione contents (GSH), and catalase (CAT) was observed following *MC* treatment. Hydroxyproline content was significantly reduced and associated morphological damages reverted to normal. The decreased expression of type III and type IV collagens was observed under immunohistochemical staining. It is concluded that *MC* fruit extract possesses antihyperglycemic, antioxidative, and cardioprotective properties which may be beneficial in the treatment of diabetic cardiac fibrosis.

## 1. Introduction

The incidence of diabetes mellitus (DM) has increased globally. Cardiovascular complications are the leading cause of morbidity and mortality among diabetic patient. Interestingly, in the absence of hypertension and coronary artery disease, there is an alteration in the morphology of the cardiac tissue. Chronic hyperglycaemic disorder in diabetic state causes glycation of body proteins and stimulates increase in free radical formation which includes reactive oxygen species (ROS) [1, 2]. Thus, there is a disturbance in the oxidant and antioxidant activities. As a consequence, complications like diabetic cardiomyopathy may develop due to the increased myocardial fibrosis and mitochondrial dysfunction [3]. The mechanism related to the diabetic cardiac disease was reported in both in vitro and in vivo studies [4].

In DM, there is the primary defect in the stimulation of glycolysis and glucose oxidation in the cardiac tissues. A major restriction to glucose utilization in the diabetic heart is the slow rate of glucose transport into the myocardium. It enhances the peripheral insulin resistance and triggers cell death [5]. DM influences the myocardium in several aspects [6]. Increased myocardial fibrosis with an increase in the deposition of connective tissues is a major finding. Accumulation of collagen in the myocardium as a result of glycosylation of lysine residues impairs the collagen degradation. As a result, fibrosis leads to injury and stiffness in the myocardial tissue [7]. It was assumed that type III and type IV collagens were mainly responsible for the increase in the deposition of connective tissue in the diabetic myocardial tissue [8]. Myocardial fibrosis attenuates the cardiac atrophy and eventually it results in cardiac failure [9]. There are reports of an increase in the connective tissue deposits in the left ventricle following induction of diabetes [9].

Dietary foods were reported to possess significant antioxidant and antidiabetic properties in diabetic state [10]. Thus, the daily intake of the antioxidant rich dietary foods improves the glycaemic status and increases the antioxidant level. This results in protection of the cardiac tissue damage [10]. Among the dietary foods,* Momordica charantia (MC)* fruit is widely consumed in south-east Asian countries like Malaysia. It belongs to the family of Cucurbitaceae, commonly known as bitter gourd or bitter melon in English and “*Peria*” in local Malaysia. To date, the effects of* MC* fruit extract on diabetic cardiac disease and myocardial fibrosis were not elucidated.

In the present study, we observed the effect of the* MC* fruit extract on cardiac fibrosis and morphological damages in the diabetic state. The antioxidant effect of* MC* fruit extract and its role in hydroxyproline in the cardiac tissues were also investigated. We also examined the antioxidant effect of* MC* fruit extract and its role in hydroxyproline in the cardiac tissues.

## 2. Methods

### 2.1. Animals

A total of thirty male Sprague-Dawley rats (weighing 250 ± 50 g) were used in the present study. Rats were placed individually in plastic cages under specific-pathogen-free conditions with a 12 h light/dark cycle. Animals were given free access to water and standard rat chow. All experimental procedures were performed according to the protocol approved by the animal ethic committee, Universiti Kebangsaan Malaysia.

### 2.2. Induction of Diabetes

Following acclimatization, all the rats were fasted 12 hrs prior to induction of diabetes. Baseline fasting blood glucose (FBG) level was recorded. Diabetes was induced in the experimental group (*n* = 18) by a single dose of streptozotocin (STZ) with a dose of 60 mg/Kg, IV (Sigma Aldrich, Germany), which was dissolved in 10 mM of normal saline. Control rats (*n* = 12) received the same volume of normal saline. Diabetes was confirmed at 72 hrs following STZ injection by measuring the FBG level. The FBG level was measured with Accu- Chek advantage II glucometer (Accu-Chek Instant, Germany). The rats with FBG level more than 8 mmol/L were labelled as diabetic [11].

### 2.3. Preparation of MC Fruit Extract by Aqueous Extraction Method

*MC* fruits (5 Kg) were purchased from the local market. The whole plant was identified by a botanist (voucher specimen—UKMB 40067). Dried fruit of* MC* underwent a reflux extraction process using 15 L water (dH_2_O) between 75 and 80°C for 3 hours. Subsequently, the extract was filtered and solidified in the −80°C refrigerator for 24 hrs. It was then freeze-dried for 3–5 days until the powder of aqueous extract was formed. In the present study, the oral dosage of 1.5 g/kg body weight was used for the treatment of control and diabetic groups [1].

### 2.4. Experimental Design

Following 4 weeks of STZ induction, the control group was equally subdivided (*n* = 6) into control group without treatment (Ctrl) and control group treated with* MC* extract 1.5 g/kg (Ctrl-*MC*). Similarly, the experimental group was further subdivided (*n* = 6) into diabetic group without treatment (DM-Ctrl), diabetic group treated with* MC* extract 1.5 g/kg (DM-*MC*), and diabetic group treated with 150 mg/kg metformin (DM-Met). The dose of metformin was adapted from a previous study [12]. Body weight and fasting blood glucose levels were measured at baseline, pretreatment, and posttreatment period. The total period of treatment for both* MC* extract and metformin was 28 days. At the end of the study, the rats were sacrificed and cardiac tissues were taken out.

### 2.5. Determination of Antioxidant Levels

Cardiac samples were homogenised in HEPES. Homogenates were centrifuged at 15,000 rpm for 5 min in a Centrikon H-401 (Germany) centrifuge at 4°C. Following centrifugation, the supernatant was collected and frozen at −30°C until analysed. All enzymatic assays were carried out at 25 ± 0.5°C using a Power Wavex microplate scanning spectrophotometer (Bio-Tek Instruments, USA) in duplicate in 96-well microplates (UV Star, Greiner Bio-One, Germany). The enzymatic reactions began with the addition of the tissue extract. The specific assay was measured.

#### 2.5.1. Measurement of Superoxide Dismutase (SOD) Activity

An amount of 20 *μ*L of the SOD standard was diluted with 1.98 mL of sample buffer (dilute); 200 *μ*L of the diluted radical detector and 10 *μ*L of standard were added per well. The reactions were initiated by adding 20 *μ*L of diluted xanthine oxidase to all the wells. Then, the 96-well plate was incubated on a shaker for 20 minutes at room temperature. The absorbance was read at 440–460 nm using plate reader. We followed an earlier protocol for the measurement of SOD activity [13].

#### 2.5.2. Measurement of Glutathione (GSH) Activity

An amount of 50 *μ*L of standard and sample were added per well in the plate. Assay cocktail was prepared by mixing the following reagents in a 20 mL vial: MES buffer (11.25 mL), reconstituted cofactor mixture (0.45 mL), reconstituted enzyme mixture (2.1 mL), water (2.3 mL), and reconstituted DTNB (0.45 mL). Then, 150 *μ*L of the freshly prepared assay cocktail was added to each of the wells containing standards and samples using a multichannel pipette. The plate was incubated on an orbital shaker. The absorbance in the wells was measured at 405–414 nm using a plate reader at five-minute intervals for a period of 30 minutes (a total of 6 measurements) [14].

#### 2.5.3. Measurement of Catalase (CAT) Activity

The reaction mixture contained 100 *μ*L of diluted assay buffer; 30 *μ*L of methanol and 20 *μ*L of the sample were added. The samples were diluted with diluted sample buffer or concentrated with an Amicon centrifuge concentrator with a molecular weight cut-off of 100,000. Reactions were initiated by adding 20 *μ*L of diluted hydrogen peroxide to all the wells. The plate was incubated on a shaker for 20 minutes at room temperature. Diluted potassium hydroxide (30 *μ*L) and catalase Purpald (Chromagen) (30 *μ*L) were added to each well. The plate was later incubated for 10 minutes at room temperature on the shaker. Then, 10 *μ*L of the catalase potassium periodate was added and incubated for 5 minutes at room temperature on a shaker. The absorbance was read at 540 nm using a plate reader. The entire procedure of determination of catalase activity was according to an earlier method described by Johansson and Borg [15].

### 2.6. Determination of Cardiac Tissue Hydroxyproline Level

Cardiac tissue was homogenized in dH_2_O, using 100 *μ*L H_2_O for every 10 mg of tissue. To 100 *μ*L sample of homogenate, 100 *μ*L concentrated HCL was added in a pressure-tight, Teflon capped vial and hydrolyzed at 120°C for 3 hours. For the reaction process, hydrolyzed sample of 10 *μ*L was transferred to a 96-well plate and evaporated to dryness under vacuum. An amount of 100 *μ*L of chloramine T reagent was added to each sample and standard and incubated at room temperature for 5 minutes. Then, 100 *μ*L of the DMAB reagent was added to each well and incubated for 90 minutes at 60°C. The absorbance at 560 nm in a microplate reader was measured [16].

### 2.7. Histological Examination

From the collected cardiac tissues, six tissues per each group were collected (2-3 mm in length) and fixed in 10% buffered formalin. Then, the tissues underwent dehydration in graded alcohol series. The tissues were cleared with xylene, embedded in paraffin, and sectioned at 5 *μ*m thickness in order to perform histological and immunohistochemical examination. Histological evaluation of ventricular cardiac tissues was performed by H&E staining, Masson's trichrome (MT), and Azan's trichrome (AT) staining. Transverse sections of cardiac tissues were captured digitally using NIH ImageJ 1.37 V. An average of 100 cells from each section were measured and mean cross-sectional cardiomyocytes were calculated under H&E stain. The ratio of blue stained connective tissue area to red stained total myocardial area was calculated under Masson trichrome stain to obtain the fraction of fibrosis (%). Both measurements were performed under image analyzer software [16, 17].

### 2.8. Immunohistochemical Examination

Regarding collagen type III and type IV expression, after perfusion and paraffin embedding, the slides were deparaffinized and rehydrated by immersing the slides through the wells: xylene (twice for 5 minutes each), 100% EtOH (twice for 5 minutes each), 95% EtOH (5 minutes), 70% EtOH (5 minutes), 50% EtOH (5 minutes), and dH_2_O for rinsed until ready for staining [18].

## 3. Results

### 3.1. Measurement of Body Weight

**Figure 1:**
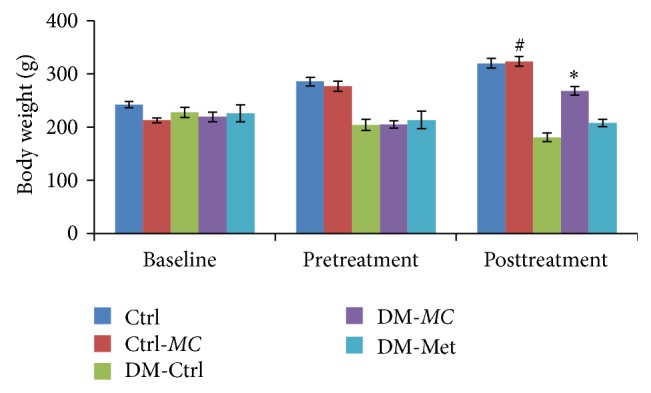
Effect of* MC* fruit extract on body weight (g) of Sprague-Dawley rats. Data were presented as means ± SEM (*n* = 6). ^*^ *P* < 0.05 versus DM-Ctrl group; ^#^ *P* > 0.05 versus Ctrl group.

The body weight of diabetic rats and nondiabetic control rats was measured (Figure 1). The weight was measured during baseline, pretreatment, and posttreatment period. At the end of pretreatment, body weight in all DM groups (DM-Ctrl, DM-*MC*, and DM-Met) was found to be reduced compared to the non-DM groups (Ctrl and Ctrl-*MC*). In posttreatment period, DM-*MC *group rats showed a significant increase (*P* < 0.05) in the body weight compared to the DM-Ctrl group. However, DM-Met group showed an increase in the body weight but it was statistically insignificant.* MC* treated control group (Ctrl-*MC*) showed no significant increase in the body weight compared to the control group (Ctrl).

### 3.2. Measurement of Fasting Blood Glucose Level

**Figure 2:**
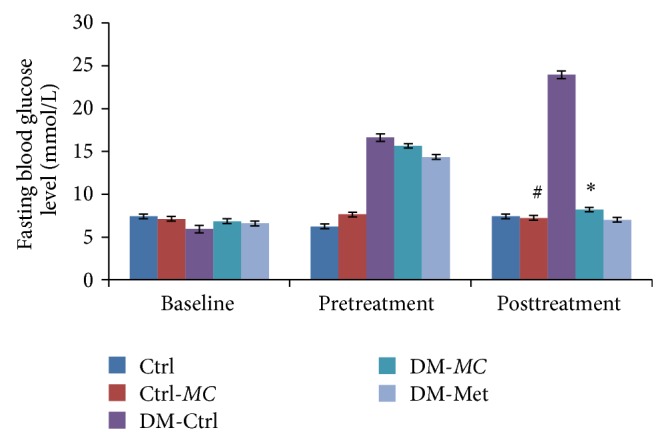
Effect of* MC* fruit extract on fasting blood glucose level (mmol/L) of Sprague-Dawley rats. Data were presented as means ± SEM (*n* = 6). ^*^ *P* < 0.05 versus DM-Ctrl group; ^#^ *P* > 0.05 versus Ctrl group.

Following 4 weeks of STZ injection, diabetic groups showed significant elevation in the FBG level compared to the control groups (Figure 2). The blood glucose level did not differ between Ctrl group and Ctrl-*MC* group. Following 8 weeks of STZ injection, DM-*MC* group and DM-Met group animals had remarkably lower fasting blood glucose levels compared to the DM-Ctrl group (*P* < 0.05). Administration with* MC* fruit extract for 28 days significantly reduced the fasting blood glucose level in diabetic rats.

### 3.3. Antioxidant Levels

At the end of the study, the animals were sacrificed with an overdose of diethyl ether. The cardiac tissue was taken out and processed for further biochemical analyses. The cardiac tissue antioxidant levels (SOD, GSH, and CAT) were measured. It was observed the antioxidant activities in cardiac tissues of DM-*MC group* rats had significantly increased compared to the DM-Ctrl group (*P* < 0.05). Statistically significant impairment in SOD, GSH, and CAT activities was observed in DM-Ctrl group rats. Surprisingly, DM-Met group rats did not show any significant changes in the antioxidant level (Table 1). Oral administration of* MC* fruit extraction diabetic rats showed a significant increase in the antioxidant enzymes.

### 3.4. Hydroxyproline Level

Cardiac tissues were homogenized to determine the hydroxyproline level using ELISA method. It was observed that DM-*MC* group rats showed significant decrease in the hydroxyproline level (*P* < 0.05) compared to the DM-Ctrl group. DM-Met group also revealed a significant decrease in the hydroxyproline level. An increase in the hydroxyproline level showed that there was an increase in the collagen deposition in the diabetic myocardial tissue. However, Ctrl-*MC* group did not show any prominent change in the hydroxyproline level compared to the Ctrl group (Table 1).

### 3.5. Histological Findings

**Figure 3:**
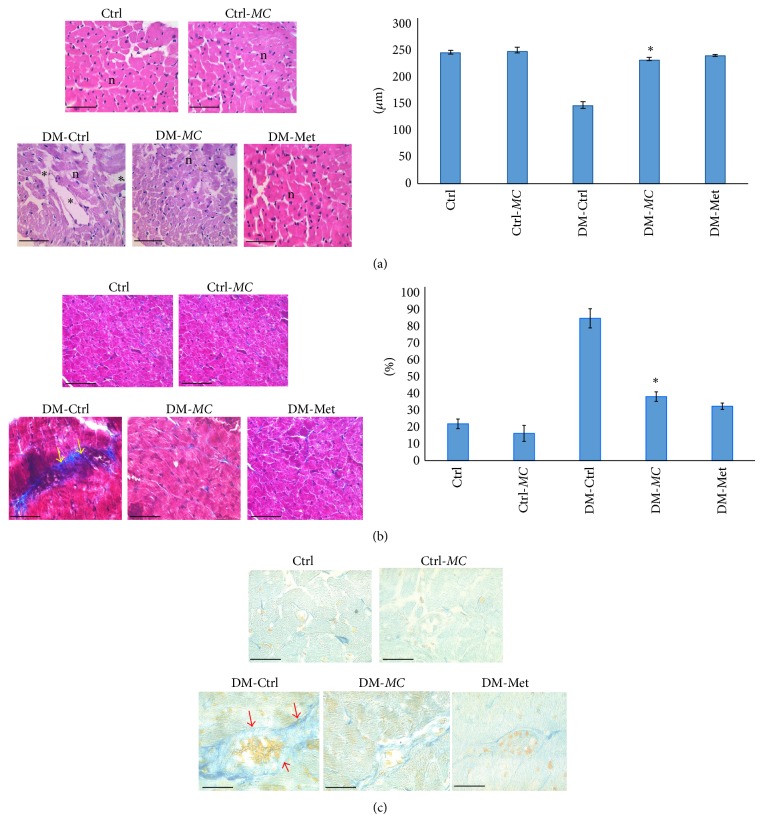
(a) Therapeutic effect of* MC* fruit extract on transverse section of left ventricular myocardial tissues of experimental rats examined by hematoxylin and eosin staining (×400, bar = 50 *μ*m). Cytoplasmic space (^*^), n = nucleus. Quantitative analysis of transverse diameter of cardiomyocyte (bar graph). ^*^ *P* < 0.05 versus DM-*MC* group. (b) Therapeutic effect of* MC* fruit extract on transverse section of left ventricular myocardial tissues of experimental rats examined by Masson trichrome staining (×400, bar = 50 *μ*m). Collagen deposition (yellow arrows, blue staining). The bar graph showing fibrosis fractions in the myocardial tissue. ^*^ *P* < 0.05 versus DM-*MC* group. (c) Therapeutic effect of* MC* fruit extract on transverse section of left ventricular myocardial tissues of experimental rats examined by Azan trichrome staining (×400, bar = 50 *μ*m). Perivascular connective tissue depositions (stained dark blue, red arrows).

Figure 3 showed histological changes in the left ventricular tissue in control and diabetic rats following 8 weeks of STZ induction. A transverse section of cardiomyocytes was observed under H&E staining. A significant decrease in the size of cardiomyocytes (*P* < 0.05) was observed in DM-*MC *group rats compared to the DM-Ctrl group (Figure 3(a)). Prominent myocardial fibrosis was observed in the cardiac tissues of DM-Ctrl group rats when viewed under MT staining. However, the amount of fibrosis was observed to be significantly reduced (*P* < 0.05) in DM-*MC *group rats compared to the DM-Ctrl group (Figure 3(b)). The increased perivascular fibrosis was observed in the cardiac tissues of DM-Ctrl group rats (Figure 3(c)). The morphological deteriorations appeared to be less in cardiac tissues of DM-*MC* group and DM-Met group of rats.

### 3.6. Immunohistochemical Staining

**Figure 4:**
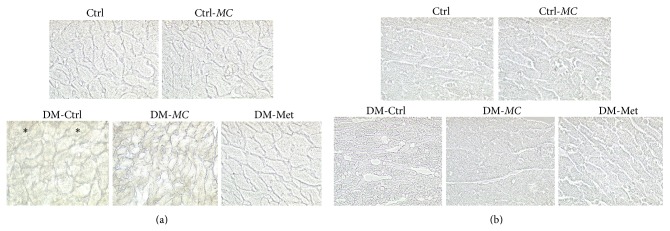
(a) Therapeutic effect of* MC* fruit extract on transverse section of left ventricular myocardial tissues of experimental rats examined by immunohistochemical staining for type III collagen expression (×400). Expression (stained light brown, ^*^). (b) Therapeutic effect of* MC* fruit extract on transverse section of left ventricular myocardial tissues of experimental rats examined by immunohistochemical staining for type IV collagen expression (×400). Expression (stained purple blue). Note: in DM-Ctrl group, the outline of cardiomyocytes is distinct from the rest of the groups.

Ventricular tissues from DM-*MC* group rats displayed patchy or scattered immunostaining for collagen type III and type IV. Increased expression of immunostaining was observed in few cardiomyocytes of the DM-Ctrl group rats (Figures 4(a) and 4(b)). Increase in type III and type IV collagens expressions indicated the severe adhesion of collagen between the myofibers. However, decreased expression of type III and type IV collagen was found in the DM-*MC* group following treatment with* MC* fruit extract. Similar findings were observed in cardiac tissues of the DM-Met group rats.

## 4. Discussion

The protective effect of* MC* fruit extract on type 1 DM has been observed earlier [19]. However, the therapeutic effect of the* MC* fruit extract of hyperglycaemia-induced cardiac fibrosis was not yet explored, to date.

Two animal studies demonstrated the potential effect of* MC* leaves extract on the diabetic cardiac tissues [19, 20]. Both studies focused on the effect of* MC* leaf extract. However, there is a paucity of experimental study with regard to* MC* fruit. To date, the definite role of* MC* fruit extract in the cardiac fibrosis in type 1 DM still remains unknown. In order to address such issues, the effect of* MC* fruit extract was studied in the animal model.

In the present study, the basic physiological, such as body weight, and biochemical, such as FBG level, parameters were measured. DM-*MC* group showed a significant increase (*P* < 0.05) in the body weight compared to DM-Ctrl group. This is in accordance with a previous study where body weight of experimental diabetic animals was found to be reduced [21]. Theoretically, the reduction in the body weight may be due to the increased catabolic rate in the state of DM. The protein degradation in the diabetic state results from unavailability of utilizing the carbohydrate as an energy source [2]. The supplementation with the extract served as a source of energy and nutrients for the body metabolic activity [22].* MC* has been shown to enhance the number of pancreatic beta cells, thus promoting the insulin secretion. Moreover, an active compound present in* MC* extract known as charantin is proven to increase GLUT4, thus increasing glucose utilization in the liver and muscle tissue [23]. The present finding with regard to body weight was comparable to DM-Met group (Figure 1). However, there was no significant difference in the body weight of Ctrl-*MC* compared to the Ctrl groups.

Besides body weight, it was found out that there was a significant decrease in the FBG in DM-*MC* group compared to DM-Ctrl group. The effect of STZ on glucose and insulin homeostasis reflects the toxin-induced abnormalities in beta cell function [24].* MC* fruit extract is enriched with flavonoid compounds such as charantins, insulin-like peptides, and alkaloids [25]. It was reported that the antihyperglycaemic activity of* MC* fruit was comparable to the usage of glibenclamide in diabetic rats [26]. Based on the present findings, it was found out that* MC* fruit extract possesses similar effect to metformin (DM-Met) in decreasing the fasting blood glucose level in diabetic rats. All the above preliminary investigations were important for the confirmation of the diabetic status.

DM is associated with an increased production of free radicals and the antioxidants defence system is impaired in chronic hyperglycaemic state [27]. Decrease in the activity of SOD, GSH, and CAT in the homogenized cardiac tissue of DM group was observed in the present study. This may be due to the increase in production of ROS that can reduce the activity of these enzymes. Following administration with the* MC* fruit extract restores the activity of the antioxidant enzymes significantly (*P* < 0.05) and may help to avoid the deleterious effects of free radicals in the diabetic state [18]. Increase in the total antioxidants properties may be due to the presence of active compounds such as charantosides in the* MC* fruit extract. However, in the present study, there was no significant increase in the antioxidant levels of Ctrl-*MC* compared to Ctrl group. In contrast to DM-*MC* group, DM-Met group did not show any significant increase in the antioxidant level in the cardiac tissues.

It was assumed that diabetic cardiac disease is probably due to an increase in collagen deposits in the myocardial tissue. In order to confirm this, we measured the hydroxyproline content in the cardiac tissues of the individual rats in the present study. DM-Ctrl group showed a significant increase (*P* < 0.05) in hydroxyproline content. Previous study demonstrated that the formation of advanced glycation end products (AGEs) in chronic hyperglycaemia accelerated the deposition of collagen and the development of myocardial stiffness in type 1 DM [28]. However, the significant decrease in hydroxyproline level was observed in the DM-*MC* group. It is in accordance with a previous study which reported that* MC* protects against the accumulation of collagen in DM animal models [29]. It was believed that an active compound of* MC*, luteolin, is responsible for reducing the collagen content in cardiac tissue [30, 31]. Similar results were noted in the DM-Met group in which hydroxyproline level was found to be reduced.

To support our assumption for collagen deposition, histological investigation was carried out. The architectural alteration in the myocardium of DM-Ctrl group of rats and reduce in transverse diameter of cardiomyocytes under H&E stain were observed in myocardial tissue of DM-*MC* group (Figure 3(a)). This is in accordance with the previous study in which the deterioration in the normal myocardium such as disorganization of myofibers, reduced cardiomyocytes transverse diameter, and scattered nuclei with increase in cytoplasmic space was detected [21]. These morphological disturbances in the cardiac tissues are associated with loss of contractile protein, myocyte dropout with interstitial fibrosis, and caloric deprivation. In our study, increased depositions of connective tissue with increased percentage of fibrotic deposition and perivascular fibrosis were observed in the DM-Ctrl group compared to the DM-*MC* group under MT and AT staining, respectively (Figures 3(b) and 3(c)). Studies showed that the increase expression of connective tissue growth factor (CTGF), transforming growth factor (TGF), and increase in collagen deposition lead to the development of fibrosis in diabetic cardiac tissue [32]. The increased glycogen levels in the diabetic state lead to the formation of perivascular fibrosis which produces the stiffness of the cardiac muscle wall [28]. These changes were found to be reduced with the administration of* MC* extract and metformin following 4 weeks of treatment. The mechanism that explains the effect of* MC* towards the diabetic cardiac disease has not been proven yet. It can be explained that the reduction in blood glucose level may increase the peripheral glucose utilization in cardiac tissue [20]. As a result, DM-*MC* group gradually restored the integrity of the connective tissue by reducing the susceptibility of the cardiovascular complications.

Although we assumed that the development of diabetic cardiac disease is most probably due to the increase deposition of collagen, it is important to ascertain the definitive collagen which is the major factors responsible for the cardiac fibrosis. Type III and type IV collagens are the important components of the extracellular matrix in the heart. Studies have shown that increased expression of type III collagen in the rat model leads to the increase risk of cardiac fibrosis and cardiac failure [33].

In the present study, DM-Ctrl group showed increased expression of type III and type IV collagens in cardiac tissue. This indicated the increased cardiac remodeling of the extracellular matrix, which leads to the impairment of cardiac tissue function. Collagen accumulation in diabetic myocardium is due to the impaired collagen degradation resulting from glycosylation of the lysine residues on collagen [34, 35].

In a study carried out by Bhoomi et al. (2013), it was demonstrated that the* MC* fruit extract reduced the cardiac fibrosis in diabetic rats [36]. It could be suggested that treatment with* MC* extract reduced the expression of type III and type IV collagen by improving the free radical scavenging activity and reducing the ROS which leads to a decrease in the oxidative stress and collagen accumulation [37]. This was also demonstrated in the present study in which DM-*MC* group showed reduced expression of type III and type IV collagen compared to the DM-Ctrl group. Metformin (DM-Met) group also proved to reduce the expression of type III and type IV collagen in myocardial tissues (Figures 4(a) and 4(b)).

## 5. Conclusion

To the best of our knowledge, the present study is the first of its kind to prove that oral administration with* MC* fruit extract could protect the cardiac remodeling in type 1 diabetic animal model by increasing the antioxidant level, reducing the collagen deposition and specific collagen expressions. Further detailed studies are needed to explore the underlying mechanism. The present study highlighted the protective effect of the* MC* fruit extract on diabetic cardiac fibrosis and encourages future research to use* MC* fruit as a supplement to treat cardiac complications arising due to diabetes mellitus.

## Figures and Tables

**Table 1:** Levels of SOD, GSH, CAT, and hydroxyproline in cardiac tissues of control and experimental groups of rats.

Groups	Ctrl	Ctrl-*MC *	DM-Ctrl	DM-*MC *	DM-Met
SOD (U/mL)	2.82 ± 0.66	3.65 ± 0.47^#^	2.30 ± 0.63	6.21 ± 0.57∗	3.62 ± 0.48
GSH (*μ*M)	516.55 ± 45.22	605.83 ± 66.16^#^	469.60 ± 70.16	1015.26 ± 70.20∗	550.56 ± 66.80
CAT (*μ*M)	42.93 ± 0.73	44.44 ± 0.49^#^	41.20 ± 0.55	48.88 ± 0.43∗	43.12 ± 0.56
Hydroxyproline (*μ*g/*μ*L)	0.0081 ± 0.0015	0.0083 ± 0.0010^#^	0.0179 ± 0.0015	0.0091 ± 0.0011∗	0.0086 ± 0.0016

Each value was expressed as mean ± SEM (*n* = 6). ^*^ *P* < 0.05 versus DM-Ctrl group; ^#^ *P* > 0.05 versus Ctrl group. SOD: superoxide dismutase activity; GSH: glutathione activity; CAT: catalase activity.
